# Determinants of COVID-19 vaccine acceptance in the Arab world: a cross-sectional study

**DOI:** 10.1186/s41256-021-00202-6

**Published:** 2021-07-12

**Authors:** M. Ihsan Kaadan, Joud Abdulkarim, Maher Chaar, Obada Zayegh, Mouhammed Ali Keblawi

**Affiliations:** 1 Department of Medicine, Boston Medical Center, Boston, Massachusetts USA; 2 Department of Medicine, Boston University School of Medicine, Boston, Massachusetts USA; 3 Faculty of Medicine, University of Aleppo, Aleppo, Syria; 4 Independent Researcher, Fort Myers, Florida USA; 5 Department of Pediatric, Aleppo University Hospital, Aleppo, Syria

**Keywords:** COVID-19, Vaccination, Vaccine hesitancy, Arab world

## Abstract

**Background:**

The Arab region is highly affected by the COVID-19 pandemic. Local governments have already started to act against the disease. However, only a few countries provided COVID-19 vaccination. Compliance with vaccination is a major topic affecting proper coverage. Thus, we aim to explore vaccine acceptance among Arab populations, and compare it with the global numbers.

**Methods:**

An internet-based survey using social media platforms was conducted, targeting adults who were able to read and understand Arabic, had access to the internet, and from all 22 Arab league countries. Due to the response rate variation between participants, the countries were grouped into four categories based on their income: Low income, Lower-middle income, Upper-middle income, and High-income economies. Data about demographics, previous COVID-19 infection, and vaccine acceptance tendency were collected and analyzed using Chi-squared (χ2) test and Logistic regression.

**Results:**

A total of 870 participants completed the survey. 59.3% of the participants were male, 53.3% were between 25 and 44 years, and 69.9% were Arabs who live inside of their home country. The COVID-19 vaccine acceptance rate was 62.4%. A significant higher tendency toward vaccination was identified in males (65.4%, *P* = 0.04) and people living outside their home countries (67.9%, *P* = 0.02). However, age group, level of education, and previous COVID-19 infection were all factors with insignificant effect. Citizens of High-income countries were more likely to accept the vaccine (70.2%).

**Conclusions:**

Less than two-thirds of Arabs are compliant with COVID-19 vaccination. This proportion is relatively lower than the global rate. It is important to develop strategies to promote vaccine acceptance and reach the ideal coverage needed to achieve efficient immunization.

## Background

Over the past decade, Arabian countries have witnessed two coronavirus related outbreaks. Following MERS-CoV-2 [1], the Novel SARS-CoV-2 (COVID-19) infection which first reported in Wuhan, China [2] has spread extensively reaching the Middle East. The earliest reports indicated the first two confirmed cases in the region were in Bahrain and United Arab Emirates (UAE) [3, 4]. In nearly 1 month, all Arab countries reported having at least one case. This includes countries that suffer from conflicts like Syria and Yemen [5]. Initially, trends of transmission and incidence were relatively low [6], nevertheless, this was attributed to lower sensitivity in detecting such newly emerging infection which resulted in a false reflection of the situation [5]. There are 4,259,756 of total confirmed COVID-19 cases and 72,950 reported deaths related to COVID-19 as of the beginning of March, 2021 [7].

Although most governments responded to the pandemic and implemented measures of protection and early warning, healthcare systems in some countries lack the proper up to date training to detect and manage the overwhelming burden of the disease [5]. The United Nations Economic and Social Commission for Western Asia (ESCWA) estimated that a loss of $42 billion could affect the Arab region due to the COVID-19, with 1.7 million job losses [5].

As of January 25, 2021, there are over 98 million cases of COVID-19 confirmed worldwide with over 2 million deaths [8]. There are 82 vaccines in development and among them 20 in Phase III [9]. Many Arab countries have started vaccinating against COVID-19, including UAE, Saudi Arabia, Bahrain, Egypt, and Jordan [10–14]. In addition, the World Health Organization (WHO) issued an emergency use for the Pfizer/BioNTech vaccine and added it to the WHO’s Emergency Use Listing (EUL) [15].

However, according to the World Health Organization (WHO), vaccine hesitancy still presents one of the top ten challenges facing the health systems around the world [16]. The World Health Organization Strategic Advisory Group of Experts (SAGE) defines vaccine hesitancy as “*delay in acceptance or refusal of vaccination despite availability of vaccination services. Vaccine hesitancy is complex and context specific, varying across time, place and vaccines. It is influenced by factors such as complacency, convenience and confidence.*” [17]. It can be related to personal beliefs, motivation, knowledge, and awareness [18]. In addition, communications between healthcare providers and vaccine recipients are critical in making a vaccination shared decision [19]. Multiple studies suggested the need to promote new policies and engage in more programs to increase vaccine acceptance [20–22]. The aim of this study is to assess and understand the COVID-19 vaccine acceptance in the Arab world, and to compare these findings with other countries.

## Methods

### Study design

This cross-sectional study was done using an anonymous internet-based questionnaire from December 26, 2020 to January 14, 2021. Recruitment was performed using targeted advertising on social media platforms (Facebook, Instagram, and Twitter). Data were collected, using Google Forms (Google, Mountain View, CA). The questionnaire distribution was based on two different sampling process: convenience and snowball sampling. Eligible participants were 18 years of age or older, from Arab League Countries, able to read and understand Arabic, and had access to the internet.

COVID-19 vaccine acceptance was assessed using established questionnaires [23–25]; however, it was modified to keep it short, concise, and easy to understand. The final questionnaire comprised two sections with a total of 9 items. The first section consists of general demographics questions, including: sex, age, education, nationality, and place of residence. Whereas the second section comprised questions related to COVID-19, such as previous exposure, family member death due to SARS-CoV-2, willingness to get a COVID-19 vaccine if available for free, and hesitancy related to the source of the vaccine.

There are 22 counties in the Arab world as defined by the World Bank [26]. These countries share common characteristics including culture, language, and religion. Due to the response rate variation between countries, the countries were grouped into four categories based on their income: Low income (Syria, Sudan, Yemen, Somalia), Lower-middle income (Algeria, Djibouti, Egypt, Morocco, Tunisia, West Bank and Gaza, Mauritania, Comoros), Upper-middle income (Iraq, Jordan, Lebanon, Libya), and High-income economies (Bahrain, Oman, Qatar, Saudi Arabia, United Arab Emirates, Kuwait) [27, 28].

### Statistical analysis

Statistical analysis was performed using Stata version 16 (StataCorp, College Station, TX: StataCorp LLC). Statistical significance was considered for a two-tailed *p* < 0.05. Frequencies and percentages were calculated for the sample demographic characteristics and COVID-19 vaccine acceptance. Chi-squared (χ^2^) test was used to analyze associations between accepting the vaccine and participants characteristics. Logistic regression was used to assess the odds ratios and their 95% confidence intervals of demographic factors and COVID-19 vaccine acceptance.

## Results

### Response rate variation between countries

Our sample comprised 870 participants from 17 countries. Response rate varied between countries; Syria 267 (30.7%), Egypt 118 (13.6%), Lebanon 107 (12.3%), Algeria 83 (9.5%), Libya 75 (8.6%), Saudi Arabia (KSA) 43 (4.9%), Iraq 39 (4.5%), Morocco 39 (4.5%), Tunisia 28 (3.2%), Kuwait 21 (2.4%), West Bank and Gaza 21 (2.4%), Yemen 21 (2.4%), Jordan 10 (1.1%), Sudan 6 (0.7%), Bahrain 2 (0.2%), Mauritania 2 (0.2%), UAE 1 (0.1%).

### Sample characteristics

Of 870 participants, 471 were males (54.1%). Age groups varied in distribution; most of the sample 464 (53.3%) were between 25 and 44 years, whereas 248 (28.5%) were among 45–64 age group, and 119 (13.7%) were 18–24 years old. Only 9 individuals (3.6%) were over 65 years (Table 1). Additionally, most individuals were college degree holders 472 (54.3%), followed by 200 (23%) graduate school degree holders, 142 (16.3%) high school degree holders, and 56 (6.4%) with no high school degree. Almost one third of the sample 262 (30.1%) were Arabs who live outside of their home country.

**Table 1:** Sociodemographic and Other Characteristics of the Study Population (*N* = 870)

Sociodemographic Characteristics		N (%)
Gender	Male	471 (54.1)
	Female	399 (45.9)
Age	18–24	119 (13.7)
	25–44	464 (53.3)
	45–64	248 (28.5)
	> 65	39 (4.5)
Education	Graduate school	200 (23.0)
	College	472 (54.3)
	High school	142 (16.3)
	No high school	56 (6.4)
Place of Residence	Inside home country	608 (69.9)
	Outside home country	262 (30.1)
Previous COVID-19 infection	Yes	170 (19.5)
	No	700 (80.5)
Family death due to COVID-19	Yes	232 (26.7)
	No	638 (73.3)
COVID-19 Vaccine Acceptance	Yes	543 (62.4)
	No	327 (37.6)

### Main findings

**Fig. 1:**
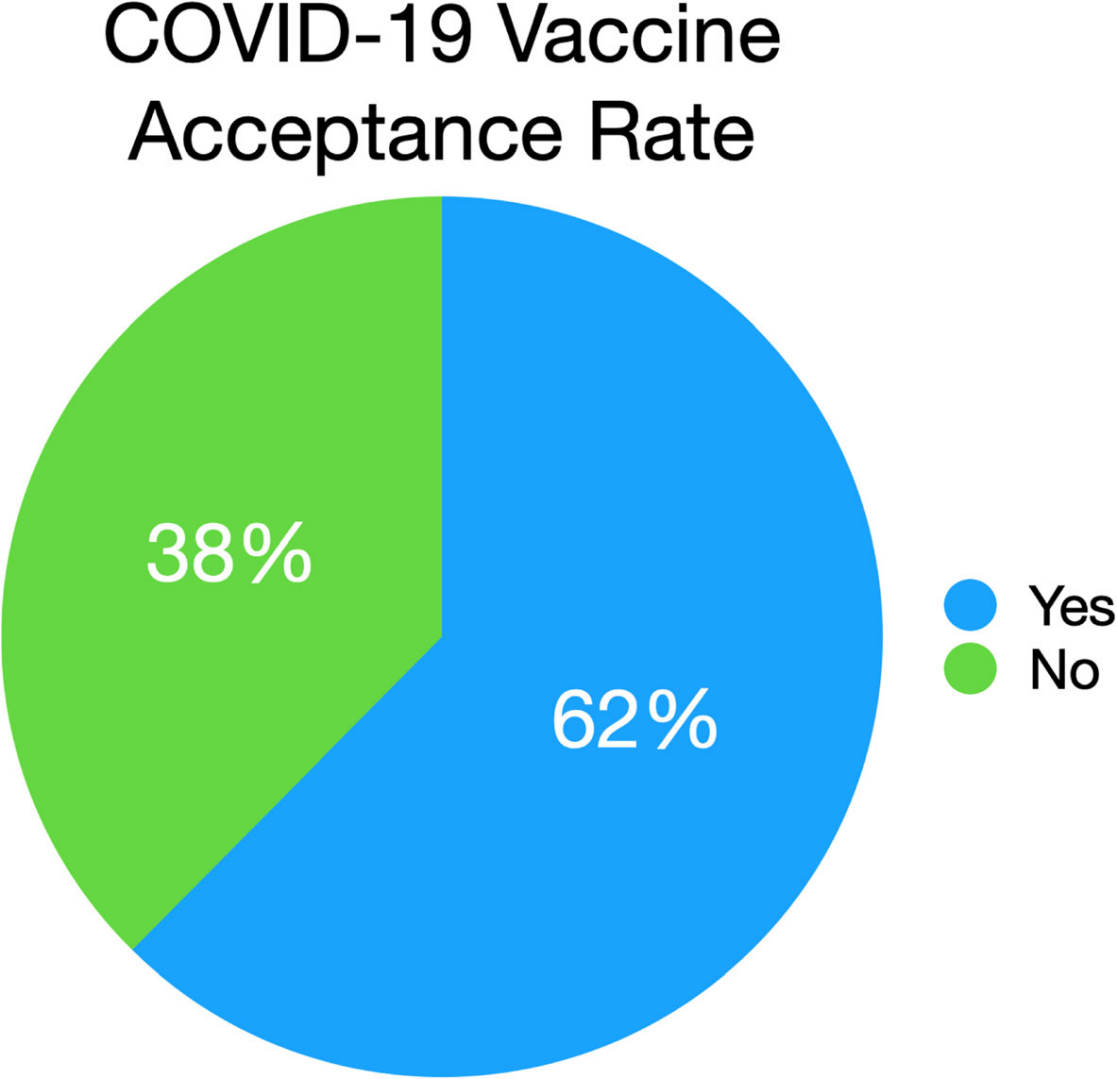
COVID-19 vaccine acceptance rate in the Arab world

During our study period, 170 (19.5%) individuals indicated that they were infected with COVID-19 before, and few more 232 (26.7%) had lost a relative or family member because of COVID-19. 543 (62.4%) of the participants would accept COVID-19 vaccine if it became available free of charge (Fig. 1). More males (65.4%) than female (58.9%) would accept getting the vaccine (*P* < 0.04) (Table 2).

**Table 2:** Bivariate Associations Between Respondents Characteristics and COVID-19 Vaccine Acceptance (*N* = 870)

Variables	COVID-19 Vaccine Acceptance n (%)		
	Yes	No	*p*-value
Gender			
Male	308 (65.4)	163 (34.6)	0.04
Female	235 (58.9)	164 (41.1)	
Age			
18–24	72 (60.5)	47 (39.5)	0.87
25–44	289 (62.3)	175 (37.7)	
45–64	159 (64.1)	89 (35.9)	
> 65	23 (59.0)	16 (41.0)	
Education			
No High school	36 (64.3)	20 (35.7)	0.13
High school	86 (60.6)	56 (39.4)	
College	297 (62.9)	175 (37.1)	
Graduate school	124 (62.0)	76 (38.0)	
Place of Residence			
Inside home country	365 (60.0)	243 (40.0)	0.02
Outside home country	178 (67.9)	84 (32.1)	
Previous COVID-19 infection			
No	445 (63.6)	255 (36.4)	0.15
Yes	98 (57.6)	72 (42.4)	
Family death due to COVID-19			
No	387 (60.7)	251 (39.3)	0.07
Yes	156 (67.2)	76 (32.8)	

The highest accepting rate is among 45–64 age group (64.1%), followed by (62.3%) and (60.5%) among 25–44 and 18–24 groups respectively (*P* < 0.87). In addition, participants with no high school found to have higher acceptance rate compared to graduate school or High school degree holders; (64.3% vs. 62.0% or 60.6% respectively) (*P* < 0.13). However, none of the last to two relationships were statistically significant. In terms of infection history, those who had no previous COVID-19 infection history had higher COVID-19 acceptance rate than those with no infection history (63.6% vs. 57.6%), however this relationship was not statistically significant (*p* < 0.15). Furthermore, no association was found between countries’ incomes and their acceptance of COVID-19 vaccine (*p* = 0.12). However, a higher rate of acceptance was noted (Table 3) in countries with upper-middle income and high-income (65.4 and 70.2%, respectively).

**Table 3:** Bivariate Associations Between Respondents Characteristics and COVID-19 Vaccine Acceptance (*N* = 870)

Variables	COVID-19 Vaccine Acceptance n (%)		
	Yes	No	*p*-value
Country Groups			
Low-Income	178 (63.3)	103 (36.6)	0.12
Lower-Middle Income	167 (57.4)	124 (42.6)	
Upper-Middle Income	151 (65.4)	80 (34.6)	
High-Income	47 (70.2)	20 (29.8)	

Binary logistic regression analysis was performed (Table 4) to predict COVID-19 vaccine acceptance among Arab countries. It was noted, in our multivariate model, that females were less likely to accept the vaccine (OR: 0.76; 95% CI: 0.57–0.99; *p* = 0.04) than males. Additionally, individuals who reside outside of their home countries found 1.41 (95% CI: 1.03–1.91; *p* = 0.02) times likely to accept COVID-19 vaccine compared with those who reside in their home countries.

**Table 4:** Binary Logistic Regression for COVID-19 Vaccine Acceptance by Respondents Characteristics. (*N* = 870)

Variables	OR	SE	95% CI	*p*-value
Gender				
Male	REF	REF	REF	
Female	0.76	0.11	0.57–0.99	0.04
Age				
18–24	REF	REF	REF	
25–44	1.07	0.22	0.71–1.62	0.72
45–64	1.16	0.26	0.74–1.82	0.50
> 65	0.93	0.35	0.44–1.95	0.86
Education				
No high school	REF	REF	REF	
High school	0.85	0.27	0.44–1.62	0.62
College	0.94	0.27	0.52–11.68	0.84
Graduate school	0.90	0.28	0.48–1.67	0.75
Place of Residence				
Inside home country	REF	REF	REF	
Outside home country	1.41	0.22	1.03–1.91	0.02
Previous COVID-19 infection				
No	REF	REF	REF	
Yes	0.77	0.13	0.55–1.09	0.15
Family death due to COVID-19				
No	REF	REF	REF	
Yes	1.33	0.21	0.96–1.82	0.07

## Discussion

The overall COVID-19 vaccine acceptance rate among Arabs who participated in this survey is 62.4%. However, this rate varied between countries’ category groups. There was a wide range of acceptance rate among participants; those who live in High-Income countries had the highest acceptance rate (70.2%), whereas those who live in Lower-Middle Income countries had the lowest acceptance rate (57.4%). This rate variation was also reported in other countries [29].

### COVID-19 vaccine acceptance rate variation between countries

The COVID-19 vaccine acceptance rate in our study is lower than the rate previously reported in China (91.3–72.6%) [30–33], Indonesia (93.3%) [34], Israel (75.0%) [35], Ecuador (97.0–71.9%) [32, 36], Malaysia (94.3%) [37], Denmark (80.0%) [38], UK (90.1–64.0%) [32, 38–42], Portugal (75.0%), Netherland (73.0%) [38], Germany (68.4–70.0%) [32, 38], Canada (68.7–80.0%) [32, 43], Turkey (69.0%) [39], Brazil (85.4%), South Africa (81.6%), South Korea (79.8%), Mexico (76.3%), India (74.5%), Spain (74.3%), Singapore (67.9%), Sweden (65.2%), Nigeria (65.2%) [32], and Australia (77.3%) [44]. However, it is higher than Poland (56.3%), Russia (54.9%) [32], and Chile (49%) [45], as reported in other studies. In addition, France (58.9–77.1%) [32, 38, 46, 47], the United States (56.9–75.4%) [25, 32, 43, 48, 49], and Italy (77.3–53.7%) [32, 38, 50] have wider range of acceptance rate than the Arab countries depending on the studies. This comparison demonstrates that the hesitancy toward COVID-19 vaccine is higher in the Arab countries compared to the majority of other parts of the world. Therefore, more studies warranted to explore further this phenomenon. In addition, regional health policy makers need to take immediate actions to reduce the disease burden in a region that has the fourth highest cases prevalence after the USA, Brazil and Russia [7]. This can be done when local governments provide free of charge vaccination, especially for those with low income to make it more acceptable [51]. Furthermore, this willingness to pay for vaccine also depends on other different factors including employment status and preexistence chronic diseases [52]. Additionally, raising the public awareness, and enhancing the knowledge about the disease risk may create more responsible behavior among the public [53]. These interventions would increase the vaccine acceptance rate.

### COVID-19 vaccine acceptance rate variation based on gender

Similar to our findings, the acceptance rate differs between gender; men are more likely to accept COVID-19 vaccine compared to women [23, 30, 39, 47]. This gender hesitancy may be explained due to potentially higher fear of injection or side effects concerns in females [54]. Another possible explanation could be related the psychological differences between both genders. For example, depression and anxiety prevalence tends to be higher in women than men [55–58]. Furthermore, the source of information might play a great role in vaccination decisions. Females seem to rely more on the internet for health-related information [59–61]. On the other hand, males tend to use the internet to communicate with their physicians more than females [59].

### COVID-19 vaccine acceptance rate variation based on place of resident

In this study, we noticed that participants who reside outside of their home countries are more likely to accept COVID-19 vaccine compared with those who reside in their home countries (OR: 1.41, 95% CI: 1.03–1.91; *p* = 0.02). Human nature tend to follow other individuals’ behavior [62]. Therefore, the acceptance rate follows the general population where the individual resides in. Furthermore, Arab world tend to be among the tightest cultures in the world, compared to other countries [63], and tight cultures are associated with higher mortality rates compared to loose cultures [64]. Therefore, these differences in human behaviors may explain the COVID-19 vaccine acceptance variation between those who reside inside their home countries and outside their home countries [65].

### Limitations

This study is a cross-sectional study and exploratory in nature, which was conducted at a specific time point. Also, we used an online questionnaire to collect our data, which may lead to selection bias and accessibility issues [66]. Another limitation is the limited sample size and the unequal distribution of participants, where the majority of subjects reside in low-income and lower-middle income countries. Finally, younger adults were predominant in the study sample and the opinion of elderly and higher risk population was limited. Therefore, generalizability of the results is precluded, and further studies are needed in the Arab world. Our study is the first of its kind, explored the determinants of COVID-19 vaccine acceptance in the Arab world, and compared the acceptance rate with other countries. This report shed the light on a new emerging challenge related to COVID-19 vaccine hesitancy in the Arab world, and its consequence on the local health systems.

## Conclusion

Our results found that less than two-third of Arabs who participated in this study would accept a COVID-19 vaccine if it becomes available. Men were more than women, and those who live abroad are more likely to accept the vaccine compared to those who live inside their home country. Additionally, acceptance rate varied between countries with individuals residing in high-income countries being the highest. Vaccination hesitancy was in-line with similar studies around the globe, but the acceptance rate in the Arab world is lower than the global rate. Therefore, strategies are needed to prompt COVID-19 vaccine locally, and new interventions shall be implemented to accomplish COVID-19 herd immunity in the Arab world.

## Data Availability

The datasets generated during and/or analyzed during the current study are available from the corresponding author on reasonable request.

## Abbreviations

MERS-CoV-2: Middle East Respiratory Syndrome Coronavirus-2
SARS-CoV-2: Severe Acute Respiratory Syndrome Coronavirus 2
COVID-19: Coronavirus Disease 2019
